# Case of Triplets

**Published:** 1845-09

**Authors:** 


*Case af Triplets.*—Dr. Parsons, of Macon, Georgia, informs the public that a Mrs. Chance, of Burke Co., had three children at one birth, lately, of the ordinary size and well developed. Two of them were united from the axilla to the upper part of the hip. The single child is living, but the two united ones died.—*Ibid.*

